# Predictors of Life Satisfaction in U.S. Adults: A Cross-Sectional Analysis of Health Status, Physical Activity, Employment, and Intersectional Factors

**DOI:** 10.7759/cureus.86872

**Published:** 2025-06-27

**Authors:** Hung Yin Lau, Sherrie M Welfel

**Affiliations:** 1 School of Health Sciences, Liberty University, Lynchburg, USA

**Keywords:** employment status, health status, interdependence, intersectionality, life satisfaction, overall well-being, physical activity

## Abstract

Introduction

Life satisfaction, a key indicator of well-being, is shaped by complex interactions between health, physical activity, employment, and demographic factors. Despite evidence linking these variables individually to well-being, their collective impact - particularly through an intersectional lens (e.g., age, sex, race) - remains inadequately studied. This study addresses gaps by analyzing how these factors jointly predict life satisfaction among U.S. adults, with implications for public health and policies.

Objectives

To investigate the relationship between self-reported overall health status, adherence to physical activity guidelines (including aerobic and strength-training exercises), employment status, and levels of life satisfaction among U.S. adults.

Methods

A secondary analysis of 2022 National Health Interview Survey (NHIS) data of 27,648 adults examined self-reported health (ranging from excellent to poor), physical activity (none, aerobic, strength, or both), employment (≥35 hours/week), and life satisfaction. Using Goodman and Kruskal’s γ, chi-square tests, and multinomial logistic regression (controlling for age, sex, race), the study assessed associations among these variables, individually and collectively, accounting for the survey's complex sampling design. The analysis adjusted for the NHIS’s stratified multistage sampling design and was performed using IBM SPSS version 26 (IBM Corp., Armonk, NY, USA), with a significance level set at α=0.05.

Results

Health status demonstrated the most significant correlation (γ=0.514, p<0.001): 68.3% of individuals in "excellent" health indicated "very satisfied," compared to only 12.3% of those in "poor" health. Adherence to physical activity guidelines also significantly influenced satisfaction levels (χ²=625.43, p<0.001); individuals who met both aerobic and strength training guidelines had twice the odds of reporting high satisfaction (OR=2.423, p=0.016). Employment showed a weak association (χ²=9.99, p=0.019), with 80.7% of those working 35 hours or more per week reporting slightly higher satisfaction levels (47% "very satisfied") compared to non-workers (45.8%). Regression analysis highlighted health (OR=4.425 for "excellent" health, p<0.001) and physical activity (OR=2.241 for strength training, p=0.027) as key predictors of satisfaction. Older adults (OR=1.022 per year, p<0.001) and males reported higher satisfaction levels, whereas females exhibited lower odds of dissatisfaction (OR=0.683, p=0.008). Although racial disparities were not statistically significant, intersectional trends hinted at complex barriers, such as limited healthcare access within marginalized communities.

Conclusion

This study highlights the significant associations between health status, physical activity, and life satisfaction among U.S. adults, with better self-reported health and higher activity levels linked to greater well-being. However, employment status showed a weaker relationship, suggesting that workplace policies should prioritize holistic well-being over mere work hours. Future research should employ longitudinal designs to establish causality and explore subgroup differences, while public health interventions should promote physical activity and preventive healthcare to enhance life satisfaction. Policymakers and employers should integrate these findings into wellness programs to foster healthier, more satisfied communities.

## Introduction

The pursuit of life satisfaction - a cognitive evaluation of one’s overall quality of life relative to personal aspirations and achievements - has emerged as a critical dimension of well-being in an aging global population [1]. In the United States, adults aged 18 and older constitute 77.9% of the population, a figure projected to grow as life expectancy increases and birth rates decline. By 2020, the median age reached 42 years, reflecting a societal shift toward prioritizing health, longevity, and fulfillment across the lifespan [2]. Life satisfaction is shaped by a complex interplay of factors, including health status, physical activity, employment, and sociodemographic characteristics. Yet existing research often examines these variables in isolation, neglecting their interconnectedness and the moderating role of intersectional identities such as race, gender, and socioeconomic status [3-5]. This fragmented approach limits the development of holistic public health strategies to address disparities in well-being, particularly among marginalized communities.

Health status consistently emerges as a cornerstone of life satisfaction, with self-reported health serving as a robust predictor of subjective well-being [6,7]. Physical activity further improves this relationship, as adherence to guidelines promoting regular aerobic and strength-training activities is linked to enhanced mental and physical health outcomes [8,9]. Employment, while traditionally viewed as a determinant of financial stability, has shown inconsistent associations with life satisfaction, suggesting that workplace environments and job quality may outweigh mere hours worked in influencing well-being [10,11]. Despite these insights, systemic inequities persist. Marginalized groups, including racial minorities and women, face disproportionate barriers to healthcare access, safe recreational spaces, and stable employment, exacerbating disparities in life satisfaction [4,12]. For instance, 75% of U.S. adults fail to meet physical activity guidelines, with lower adherence rates observed in low-income and minority communities, underscoring the urgency of addressing structural determinants of health [8,13,14].

The World Health Organization (WHO) defines health as a state of complete physical, mental, and social well-being, yet this ideal remains elusive for many [15]. Intersectionality - a framework that examines how overlapping social identities and systemic inequities shape lived experiences - provides a critical lens for understanding disparities in life satisfaction [4,5]. While intersectional approaches have gained traction in sociological and public health research, their application to quantitative studies of well-being remains limited, particularly in large-scale national surveys [4,16]. This gap hinders efforts to design targeted interventions that address the unique challenges faced by individuals navigating multiple axes of marginalization.

This study leverages data from the 2022 National Health Interview Survey (NHIS) to explore the relationships between health status, physical activity, employment, and life satisfaction among U.S. adults, while centering intersectionality as a guiding framework. The NHIS, a nationally representative survey administered by the National Center for Health Statistics (NCHS), employs a stratified multistage sampling design to capture health-related data from non-institutionalized civilians [17]. By integrating demographic variables such as age, sex, and race/ethnicity into analyses, this research aims to illuminate how intersecting identities mediate access to resources and opportunities that foster well-being.

Despite extensive literature on individual determinants of life satisfaction, few studies adopt a comprehensive approach to examine their collective impact. Intersectionality, a framework introduced by Crenshaw [16], emphasizes how overlapping social identities (e.g., race, gender, class) compound systemic inequities, shaping access to resources that influence well-being. For instance, marginalized groups often face barriers to healthcare and employment stability, exacerbating disparities in life satisfaction [4,12]. Existing research often overlooks the compounding effects of socioeconomic disadvantage, geographic disparities, and cultural norms, particularly in diverse populations [10,12]. Furthermore, the reliance on cross-sectional data in many studies limits causal inference and obscures temporal trends in well-being [18]. This study addresses these gaps by employing robust statistical methods to analyze synergistic relationships between predictors while advocating for longitudinal designs in future research.

The findings of this study hold significant implications for public health policy, workplace wellness programs, and community-based interventions. By identifying key predictors of life satisfaction and highlighting disparities across demographic groups, this research provides a foundation for equitable strategies to enhance well-being in an increasingly diverse and aging population. Policymakers, healthcare providers, and employers are urged to prioritize preventive care, expand access to recreational resources, and foster inclusive environments that support holistic health.

## Materials and methods

Study design and setting

This cross-sectional study utilized publicly available data from the 2022 NHIS, a nationally representative household survey conducted annually by the NCHS [2]. The NHIS employs a stratified multistage sampling design to collect health-related data from non-institutionalized U.S. civilians, excluding individuals in long-term care facilities, military personnel, and incarcerated populations [17]. Data collection occurred year-round (January-December 2022) via computer-assisted personal interviewing (CAPI) using Blaise software, which enforced skip patterns, range checks, and real-time error corrections to minimize data entry inaccuracies [19]. Interviewers underwent rigorous training, including annual refresher courses on NHIS protocols, question phrasing, and confidentiality requirements. The final analytic sample included 27,648 adults, with a household response rate of 54.4% and a sample adult response rate of 47.7%, consistent with pre-pandemic NHIS benchmarks [17]. Data were anonymized, and weights were applied to account for non-response and complex survey design, ensuring nationally representative estimates. Ethical approval for secondary data analysis was obtained from the Institutional Review Board (IRB-FY23-24-1680), adhering to confidentiality protocols under Section 308(d) of the Public Health Service Act [17].

Participants

This study utilized secondary data from the 2022 NHIS, which divides the U.S. into 1,689 geographic clusters (primarily counties or groups of counties) and selects households through a dual-frame approach: 89% from address-based vendor lists (unit frame) and 11% via field enumeration in areas with limited coverage (area frame) [17]. Each cluster contained approximately 2,500 addresses, with oversampling in the 10 least populous states and Washington, D.C., to ensure geographic diversity. Households were randomly selected, and one adult (≥18 years) per household was chosen as the "sample adult" for detailed health interviews. Proxy responses were permitted only for individuals with physical or mental incapacities (n=488, 1.8% of the sample). Participants with missing responses for key variables (health status, physical activity, employment status, or life satisfaction) were excluded (n=3, non-response rate <1%). Demographic characteristics in Table 1 were representative of the U.S. adult population: 45.6% male, 54.4% female, 78% White, 12.3% Black/African American, and 6.4% Asian. Age ranged from 18 to ≥85 years (mean = 42 years) [17].

**Table 1:** Count and Frequency Percentages for Demographic, Dependent, and Independent Variables of the Study Population Summary of the variables used in the analysis along with the count and percentage frequencies for each category for sex, age, race, general health status, life satisfaction, physical activity, and employment within the population under study. AIAN: American Indian/Alaska Native

Category	Group	Count	% Frequency
Sex	Male	12598	45.6%
	Female	15050	54.4%
Age	18-84 years	26585	96.4%
	85+ years	1002	3.6%
Single and Multiple-Race Groups	White only	20474	78.0%
	Black/African American only	3231	12.3%
	Asian only	1688	6.4%
	AIAN only	269	1.0%
	AIAN and any other group	214	0.8%
	Other Single and Multiple Races	358	1.4%
General Health Status	Excellent	5659	20.5%
	Very Good	9469	34.3%
	Good	8269	29.9%
	Fair	3220	11.6%
	Poor	1028	3.7%
Life Satisfaction/Dissatisfaction	Very Satisfied	12298	44.6%
	Satisfied	14046	50.9%
	Dissatisfied	966	3.5%
	Very Dissatisfied	265	1.0%
Physical Activity Met Guidelines for Aerobic and/or Strengthening Activity	Meets Neither Criteria	12475	47.1%
	Meets Strength Only	1733	6.5%
	Meets Aerobic Only	6125	23.1%
	Meets Both Criteria	6161	23.3%
Usually Work 35+ Hours Per Week	Yes	12381	80.7%
	No	2957	19.3%

Coding and scoring criteria

To enhance transparency, the operational definitions for key variables were as follows.

Health Status

Self-reported health was coded on a 5-point ordinal scale: 1 = Excellent; 2 = Very good; 3 = Good; 4 = Fair; 5 = Poor.

Physical Activity

Participants were categorized into four mutually exclusive groups based on 2018 U.S. Department of Health and Human Services guidelines [9]: Non-adherent: Failing to meet either aerobic or strength-training guidelines; Aerobic-only adherent: Reporting ≥150 minutes/week of moderate-intensity or ≥75 minutes/week of vigorous-intensity aerobic activity; Strength-only adherent: Reporting muscle-strengthening activities two or more days/week; Fully adherent: Meeting both aerobic and strength-training guidelines.

Employment Status

Employment status was dichotomized as working ≥35 hours/week (coded as 1) or working <35 hours/week or unemployed (coded as 0).

Life Satisfaction

Life satisfaction was measured on a 4-point Likert scale: 1 = Very satisfied; 2 = Satisfied; 3 = Dissatisfied; 4 = Very dissatisfied

Exposure

The study examined three key predictors using standardized operational definitions. Health status was evaluated through a single-item self-assessment ("Would you say your health in general is excellent, very good, good, fair, or poor?"), coded on a 5-point ordinal scale (1=excellent to 5=poor) [17]. Physical activity classification followed U.S. Department of Health and Human Services guidelines [9], with participants categorized into four mutually exclusive groups: (1) non-adherent (meeting neither aerobic nor strength guidelines), (2) aerobic-only adherent (≥150 minutes/week moderate-intensity activity), (3) strength-only adherent (two or more days/week muscle-strengthening activities), and (4) fully adherent (meeting both criteria). Employment status was operationalized as a dichotomous variable (working ≥35 vs. <35 hours/week) based on standard full-time employment thresholds [17]. These measures were selected to align with established public health surveillance methodologies while maintaining conceptual clarity for cross-study comparisons.

Covariates

Analyses were adjusted for sociodemographic variables, including age, sex, and race/ethnicity, to account for their potential confounding effects on life satisfaction. Age was modeled as a continuous variable (18-99 years) to capture its linear association with life satisfaction, avoiding artificial categorization while reflecting gradual changes in well-being across adulthood [20]. Sex was dichotomized as male or female, with female as the reference category, based on self-reported gender identity to address documented disparities in health and life satisfaction between genders [21]. Race/ethnicity was categorized into five groups: White (reference), Black/African American, Asian, American Indian/Alaska Native, and Other (including mixed race and unspecified), consistent with NHIS classifications [17]. This categorization aimed to explore racial/ethnic disparities in life satisfaction, as systemic inequities often disproportionately affect minority populations in access to healthcare, employment, and recreational resources [6].

Statistical analysis

Statistical analysis began with a priori power calculation using G*Power 3.1 [22], which determined a minimum sample size of 369 for regression analyses (α = 0.05, power = 0.95) based on detecting medium effect sizes (OR = 3.47) observed in comparable studies [17]. The analysis was performed using IBM SPSS version 26 (IBM Corp., Armonk, NY, USA) [23], with a significance level set at α=0.05. Descriptive statistics summarize demographic characteristics and outcome distributions across the study population. Bivariate analyses employed Goodman and Kruskal’s γ [22] to assess ordinal associations between health status and life satisfaction, selected for its suitability in analyzing tied ranks within Likert-scale responses. Chi-square tests evaluated categorical relationships between physical activity categories, employment status, and life satisfaction. Multinomial logistic regression models were then applied to examine the collective influence of health status, physical activity, and employment on life satisfaction, adjusting for age (continuous), sex (reference: female), and race/ethnicity (reference: White). Prior to analysis, missing data were evaluated using the following protocols. Missing values were flagged using NHIS response codes (e.g., "Refused" (777), "Not Ascertained" (555)). Cases with missing key variables (health status, physical activity, employment, life satisfaction; n=3, <1%) were excluded pairwise to preserve sample size and analytical rigor. For all analyses, statistical significance was set at p < 0.05. Bias sensitivity analyses confirmed that excluded cases did not differ demographically from the analytic sample (all p>0.05 via chi-square tests). Multiple imputation was deemed unnecessary due to NHIS analytic guidelines treating non-responses as true missing [17].

Validation and reliability

The NHIS instrument exhibits strong reliability, evidenced by test-retest concordance rates surpassing 85% for self-reported variables [20]. The classification of physical activity is consistent with the criteria established by the CDC’s Behavioral Risk Factor Surveillance System (BRFSS) [13] and has been validated against accelerometer data [24]. Comprehensive interviewer training and the implementation of computer-assisted personal interviewing (CAPI) error checks have significantly reduced the potential for data inaccuracies [20].

Ethical considerations

The NHIS complies with Section 308(d) of the Public Health Service Act, which prohibits the disclosure of identifiable information [17]. Data have been de-identified, and geographic identifiers have been suppressed. Informed consent procedures adhered to the protocols established by the NCHS.

## Results

Table 1 provides an overview of self-reported health status, perceived life satisfaction, physical activity and employment status among participants. With respect to the health status the majority rated their health as excellent (20.5%), very good (34.3%), or good (29.9%), whereas a smaller group reported fair (11.6%) or poor (3.7%) health. Life satisfaction levels were notably high, with 44.6% of individuals indicating they were very satisfied and 50.9% indicating they were satisfied; only a small percentage expressed dissatisfaction (3.5%) or were very dissatisfied (1%). Physical activity levels varied, revealing that nearly half of the participants (47.1%) did not meet guidelines for either aerobic or strengthening activities. Approximately 23.1% met only the aerobic guidelines, 6.5% met only the strengthening guidelines, and 23.3% satisfied both criteria. Regarding employment, a substantial majority (80.7%) reported working 35 or more hours per week, while the remaining 19.3% did not.

Health status and life satisfaction

A robust positive association emerged between health status and life satisfaction during our investigation. To investigate the relationship between health status and life satisfaction of adults in the U.S., Goodman and Kruskal’s γ was conducted. The results of Goodman and Kruskal’s γ test in Table 2 showed a highly statistically significant association between health status and life satisfaction, γ = .514, p < .001, hence the null hypothesis was rejected. The medium γ value and the extremely small p-value (less than 0.001) provide moderate significant statistical evidence of an association between the two variables, health status and life satisfaction.

**Table 2:** Goodman and Kruskal’s γ Test Results for General Health Status and Life Satisfaction Association Among U.S. Adults Goodman and Kruskal’s γ test examining the association between general health status and life satisfaction among U.S. adults revealed a significant association (p < .001) for the sample of N=27,570 valid cases. a. Not assuming the null hypothesis. b. Using the asymptotic standard error assuming the null hypothesis.

Measure	Value	Asymptotic Standard Error^a^	Approximate T^b^	Approximate Significance (p)
Gamma (γ)	.514	.007	66.366	.000
N of Valid Cases	27570			

The cross-tabulation of the data in Table 3 revealed a clear pattern: individuals with better self-reported general health status tend to report higher levels of life satisfaction, while those with poorer health status tend to report lower levels of life satisfaction. Specifically, among adults who reported excellent health, a substantial majority (68.3%) were very satisfied with life, and only a small fraction (1.1%) expressed dissatisfaction or very dissatisfaction. In contrast, for those who reported poor health, only 12.3% were very satisfied with life, while a substantial proportion (32.1%) were dissatisfied or very dissatisfied. 

**Table 3:** Crosstabulation for General Health Status and Life Satisfaction Association Among U.S. Adults Crosstabulation for the association between general health status and life satisfaction among U.S. adults for the sample of N=27,570 respondents. Percentages within rows reflect the proportion of respondents in each life satisfaction category relative to their general health status.

General Health Status		Life Satisfaction/Dissatisfaction				Total
		Very Satisfied	Satisfied	Dissatisfied	Very Dissatisfied	
Excellent	Count	3857	1733	41	20	5651
	% within General Health Status	68.3%	30.7%	0.7%	0.4%	100.0%
Very Good	Count	5060	4228	133	38	9459
	% within General Health Status	53.5%	44.7%	1.4%	0.4%	100.0%
Good	Count	2603	5324	257	55	8239
	% within General Health Status	31.6%	64.6%	3.1%	0.7%	100.0%
Fair	Count	649	2195	300	60	3204
	% within General Health Status	20.3%	68.5%	9.4%	1.9%	100.0%
Poor	Count	125	565	235	92	1017
	% within General Health Status	12.3%	55.6%	23.1%	9.0%	100.0%
Total	Count	12294	14045	966	265	27570
	% within General Health Status	44.6%	50.9%	3.5%	1.0%	100.0%

**Figure 1:**
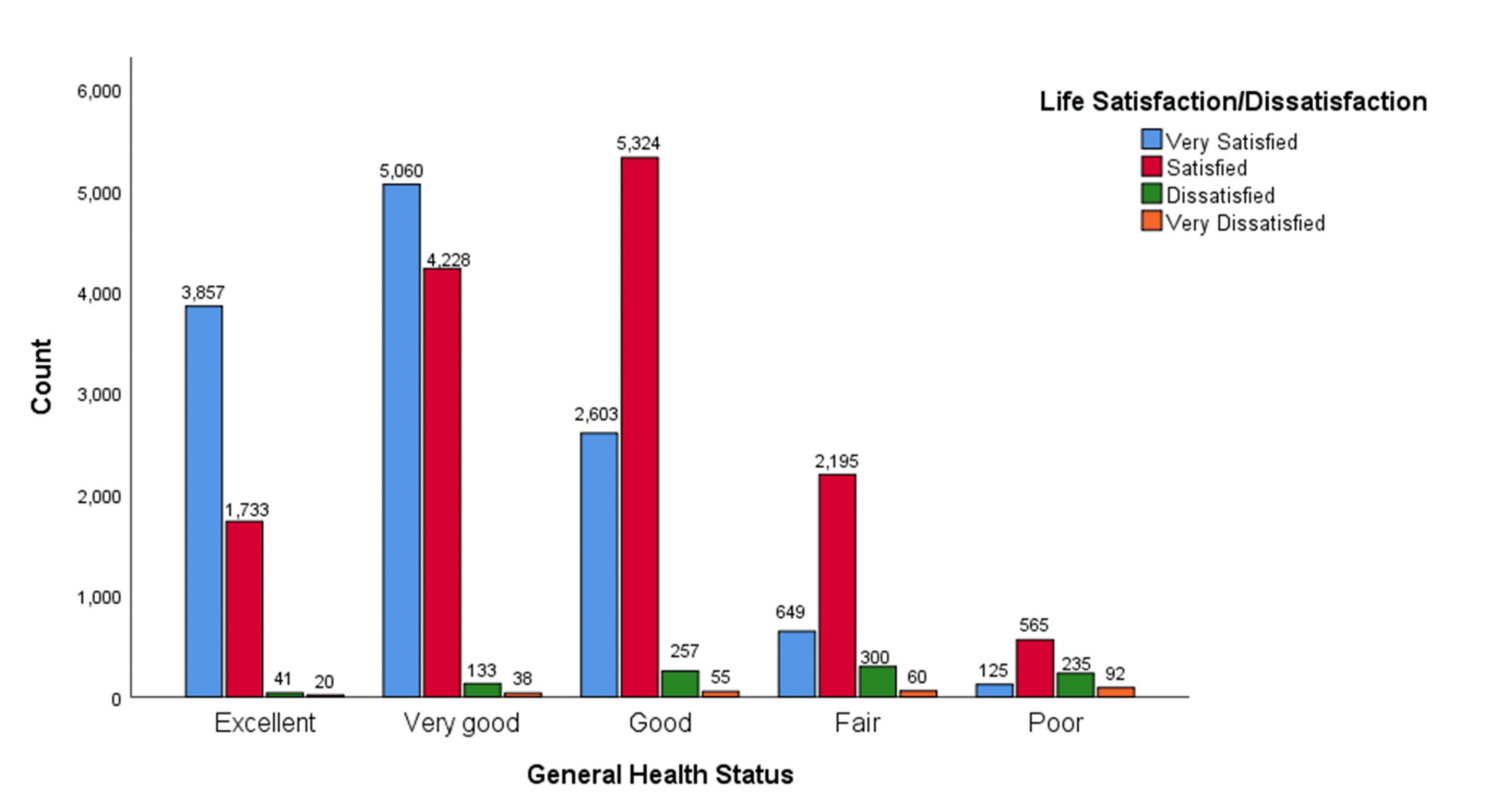
General Health Status and Life Satisfaction Association Among U.S. Adults A bar chart illustrating the relationship between general health status and life satisfaction levels among U.S. adults showed that individuals with better health status tend to report higher levels of life satisfaction.

The trend observed in the above crosstabulation is evident across all health status categories, as shown in Figure 1. Individuals in excellent health reported the highest levels of life satisfaction, with many indicating they were very satisfied. Those with very good health maintained a high satisfaction rate, while the good health category saw more individuals satisfied than very satisfied. In the fair health category, the majority were very satisfied, but this was lower than in the previous groups. Conversely, only a small fraction of individuals in poor health reported feeling very satisfied, with a notable increase in dissatisfaction as health declined. Overall, as health status improved from poor to excellent, the proportion of individuals reporting high life satisfaction increased, while those indicating dissatisfaction decreased, highlighting a consistent and clear pattern. 

Physical activity and life satisfaction

A strong association was identified between physical activity and life satisfaction in adults across the U.S. To assess the relationship between these two variables, a Chi-square test of association was utilized. The findings presented in Table 4 indicate a highly significant correlation between physical activity and life satisfaction, leading to the rejection of the null hypothesis. The substantial Chi-square statistic, combined with an exceptionally low p-value (less than 0.001), offers compelling statistical evidence to refute the null hypothesis and support the alternative hypothesis, confirming that physical activity is indeed related to life satisfaction.

**Table 4:** Chi-Square Test Results for Physical Activity and Life Satisfaction Association Among U.S. Adults Chi-Square test of association conducted to examine the association between physical activity and life satisfaction among U.S. adults showed significant relationship (p < .001) for the sample of N=26,434 valid cases. a. 0 cells (.0%) have expected count less than 5. The minimum expected count is 16.36.

Test	Value	df	Asymptotic Significance (p)
Pearson Chi-Square	625.429^a^	9	.000
Likelihood Ratio	633.543	9	.000
Linear-by-Linear Association	607.758	1	.000
N of Valid Cases	26434		

A significant negative correlation was identified between physical activity and life satisfaction among adults in the U.S., as indicated by Spearman’s rank correlation analysis (r(9) = -0.150, p < 0.001), as presented in Table 5. The findings suggest that individuals who did not engage in physical activities meet aerobic and strengthening criteria reported lower levels of life satisfaction compared to those who did meet both standards. The highly significant p-value (p < 0.001) reinforces the reliability of this result, pointing to a potentially unexpected inverse relationship between physical activity levels and subjective life satisfaction. 

**Table 5:** Correlation Test Results for Physical Activity and Life Satisfaction Association Among U.S. Adults Pearson's r and Spearman correlation are reported indicating a significant negative correlation and small effect size between physical activity and life satisfaction among U.S. adults showed significant relationship (p < .001) for the sample of N=26,434 valid cases. a. Not assuming the null hypothesis. b. Using the asymptotic standard error assuming the null hypothesis. c. Based on normal approximation.

Measures	Value	Asymptotic Standard Error^a^	Approximate T^b^	Approximate Significance (p)
Pearsons R	-.152	.006	-24.941	.000^c^
Spearman Correlation	-.150	.006	-24.676	.000^c^
N of Valid Cases	26434			

The analysis of the data presented in Table 6 indicates a distinct relationship between adherence to physical activity guidelines and levels of life satisfaction. Individuals who followed both the aerobic and strengthening activity recommendations reported notably higher life satisfaction, with 54.5% expressing that they were very satisfied, and only 2.3% indicating dissatisfaction. In contrast, among those who did not meet either guideline, only 37.9% reported being very satisfied, while a larger proportion, 6.2%, were dissatisfied or very dissatisfied. This pattern clearly illustrates the positive correlation between physical activity and life satisfaction levels. 

**Table 6:** Crosstabulation for Physical Activity and Life Satisfaction Association Among U.S. Adults Crosstabulation for the association between physical activity (meeting aerobic and/or strengthening guidelines) and life satisfaction among U.S. adults for the sample of N=26,434 valid respondents. Percentages within rows reflect the proportion of respondents in each life satisfaction category relative to their physical activity meeting guidelines for aerobic and/or strengthening activity.

Physical Activity Met Guidelines for Aerobic and/or Strengthening Activity		Life Satisfaction/Dissatisfaction				Total
		Very Satisfied	Satisfied	Dissatisfied	Very Dissatisfied	
Meets Neither Criteria	Count	4712	6943	603	175	12433
	% within Physical Activity Met Guidelines for Aerobic and/or Strengthening Activity	37.9%	55.8%	4.8%	1.4%	100.0%
Meets Strength Only	Count	759	904	59	8	1730
	% within Physical Activity Met Guidelines for Aerobic and/or Strengthening Activity	37.9%	52.3%	3.4%	0.5%	100.0%
Meets Aerobic Only	Count	3013	2920	143	42	6118
	% within Physical Activity Met Guidelines for Aerobic and/or Strengthening Activity	49.2%	47.7%	2.3%	0.7%	100.0%
Meets Both Criteria	Count	3354	2660	114	25	6153
	% within Physical Activity Met Guidelines for Aerobic and/or Strengthening Activity	54.5%	43.2%	1.9%	0.4%	100.0%
Total	Count	11838	13427	919	250	26434
	% within Physical Activity Met Guidelines for Aerobic and/or Strengthening Activity	44.8%	50.8%	3.5%	0.9%	100.0%

**Figure 2:**
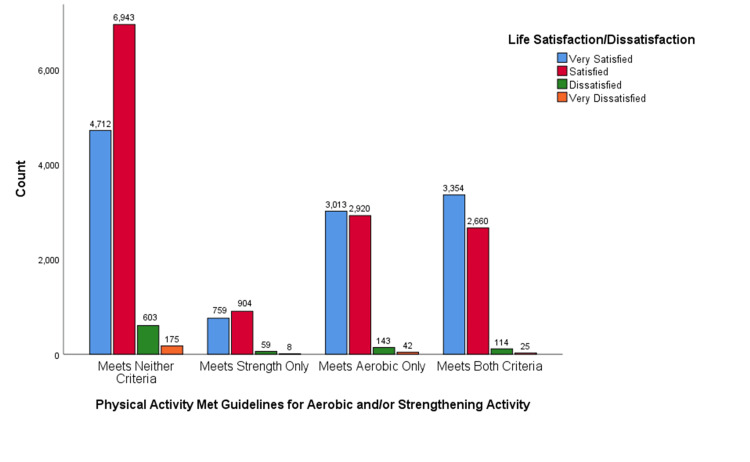
Physical Activity and Life Satisfaction Association Among U.S. Adults A bar chart illustrated the relationship between physical activity and life satisfaction levels among U.S. adults which showed that individuals meeting both aerobic and/or strengthening guidelines criteria tend to report higher levels of life satisfaction following people meeting neither criterion.

The pattern observed in the above crosstabulation holds true across varying levels of physical activity, as illustrated in Figure 2. Among individuals who did not adhere to either of the physical activity recommendations, a considerable portion reported being very satisfied with their lives. As we consider those meeting only the strength criteria, the proportion of individuals expressing high satisfaction remained stable. In contrast, among those who met solely the aerobic guidelines, there was an uptick in life satisfaction; however, this was still lower than those fulfilling both strength and aerobic criteria. The group that achieved both sets of guidelines witnessed an increase in individuals reporting very high life satisfaction compared to those meeting just one. Overall, with increased physical activity levels, a greater percentage of individuals reported feeling very satisfied with life, while dissatisfaction notably decreased from the group not meeting criteria to those meeting both criteria.

Employment status and life satisfaction

A Chi-square test of association was performed to explore the connection between employment status and life satisfaction among U.S. adults, with the results detailed in Table 7 revealing a statistically significant relationship between working 35 or more hours per week and life satisfaction (χ²(3) = 9.988, p = 0.019). This indicates that the null hypothesis can be rejected, as the p-value of 0.019 is below the traditional threshold of 0.05. Nevertheless, the Chi-square value of 9.988 suggests a relatively weak association between these two variables, indicating that while the relationship is significant, its strength may not be particularly robust.

**Table 7:** Chi-Square Test Results for Employment Status and Life Satisfaction Association Among U.S. Adults Chi-Square test of association conducted to examine the association between employment status and life satisfaction among U.S. adults showed significant relationship (p = .019) for the sample of N=15,310 valid cases.

Test	Value	df	Asymptotic Significance (p)
Pearson Chi-Square	9.988^a^	3	.019
Likelihood Ratio	9.351	3	.025
Linear-by-Linear Association	4.299	1	.038
N of Valid Cases	15310		

Spearman’s rank correlation analysis was conducted to explore the association between regularly working 35 or more hours per week and life satisfaction among U.S. adults. As outlined in Table 8, the results indicated a slight positive correlation, r(9) = .013, p = 0.107, yet this finding was not statistically significant. Therefore, while there may be some degree of relationship between the number of hours worked and life satisfaction, the evidence is insufficient to assert that employment status plays a meaningful role in influencing life satisfaction within the sample examined. 

**Table 8:** Correlation Test Results for Employment Status and Life Satisfaction Association Among U.S. Adults Pearson's r and Spearman correlation reported a nonsignificant correlation between employment status and life satisfaction among U.S. adults (p > .05) for the sample of N=15,310 valid cases. a. Not assuming the null hypothesis. b. Using the asymptotic standard error assuming the null hypothesis. c. Based on normal approximation.

Measures	Value	Asymptotic Standard Error^a^	Approximate T^b^	Approximate Significance (p)
Pearsons R	.017	.008	2.074	.038^c^
Spearman Correlation	.013	.008	1.612	.107^c^
N of Valid Cases	15310			

An examination of the data in Table 9 reveals notable differences in life satisfaction levels among individuals who typically work 35 or more hours per week compared to those who do not. Specifically, 47.0% of those in the 35+ hour group reported being very satisfied, slightly surpassing the 45.8% of their counterparts who work fewer hours. Furthermore, the 35+ hour workers displayed lower dissatisfaction levels, with only 2.0% expressing dissatisfaction and 0.4% indicating they were very dissatisfied, compared to 2.8% and 0.6% among those who do not usually work 35 hours or more. This data suggests a slight correlation between longer working hours and increased life satisfaction.

**Table 9:** Crosstabulation for Employment Status and Life Satisfaction Association Among U.S. Adults Crosstabulation for the association between employment status (Usually Work 35+ Hours Per Week) and life satisfaction among U.S. adults for the sample of N=15,310 valid respondents. Percentages within rows reflected the proportion of respondents in each life satisfaction category relative to their employment status.

Usually Work 35+ Hours Per Week		Life Satisfaction/Dissatisfaction				
		Very Satisfied	Satisfied	Dissatisfied	Very Dissatisfied	Total
Yes	Count	5804	6255	247	52	12358
	% within Usually Work 35+ Hours Per Week	47.0%	50.6%	2.0%	0.4%	100.0%
No	Count	1352	1499	82	19	2952
	% within Usually Work 35+ Hours Per Week	45.8%	50.8%	2.8%	0.6%	100.0%
Total	Count	7156	7754	329	71	15310
	% within Usually Work 35+ Hours Per Week	46.7%	50.6%	2.1%	0.5%	100.0%

**Figure 3:**
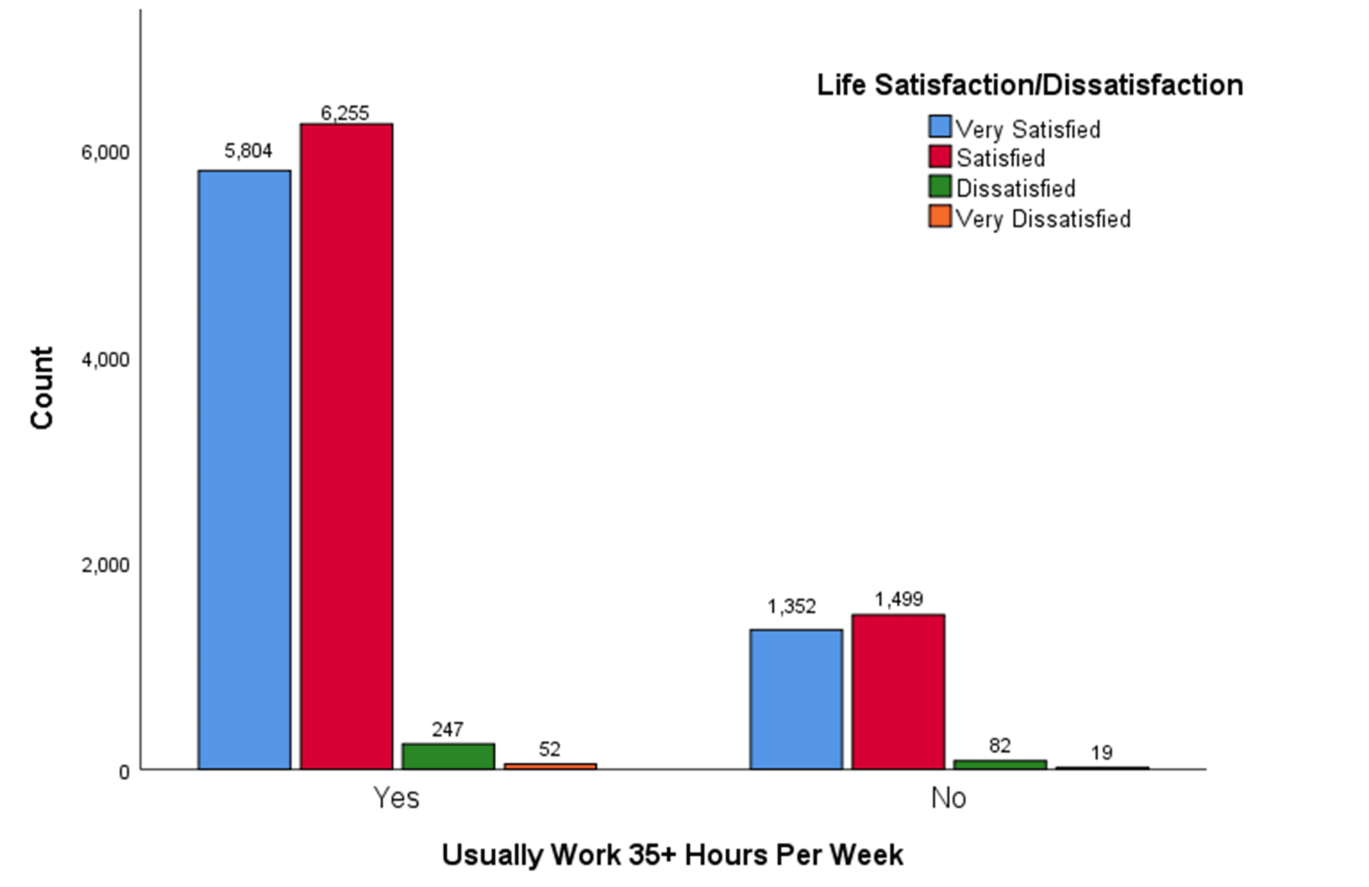
Employment Status and Life Satisfaction Association Among U.S. Adults A bar chart illustrated the relationship between the life satisfaction levels of U.S. adults based on whether they usually work 35+ hours per week. Those who work 35+ hours per week reported higher levels of life satisfaction which suggested that working longer hours may be associated with increased life satisfaction among U.S. adults.

The pattern observed in the above crosstabulation is reflected across various levels of life satisfaction, as depicted in Figure 3, where individuals typically working 35 or more hours per week exhibit a range of contentment levels. Within this demographic, a notable proportion reported being very satisfied with their lives, with a relatively higher number expressing overall satisfaction. In contrast, fewer individuals indicated dissatisfaction, and an even smaller segment reported feeling very dissatisfied. When compared to those not regularly working 35 hours or more, there are fewer individuals claiming to be very satisfied or satisfied in this latter group, with similar trends emerging in the dissatisfaction categories. Overall, the data reveal a clear trend: as work hours increase, there is a corresponding rise in the number of individuals expressing high life satisfaction and a decline in those reporting dissatisfaction or very dissatisfaction.

Multivariate analysis

The final multinomial logistic regression model, as detailed in Table 10 and controlling for age, sex, and race, revealed that health status and physical activity were the strongest predictors of life satisfaction. Age served as a moderator, with each additional year increasing the odds of being "very satisfied" by 2.2% (OR = 1.022, 95% CI: 1.014-1.030, p < 0.001). Females exhibited lower odds of dissatisfaction compared to males (OR = 0.730, 95% CI: 0.565-0.944, p = 0.017); however, the analysis showed no significant gender difference in "very satisfied" responses (p = 0.140). Race/ethnicity did not independently predict satisfaction (p > 0.05). The regression analysis confirmed that individuals in "excellent" health had 4.4 times higher odds of reporting "very satisfied" compared to those in "poor" health (OR = 4.425, 95% CI: 3.871-5.058, p < 0.001), as illustrated in Table 10. Moreover, meeting both physical activity guidelines was identified as a key predictor, resulting in 2.4 times higher odds of high satisfaction (OR = 2.423, 95% CI: 1.180-4.974, p = 0.016). Additionally, adherence to aerobic-only guidelines was linked to increased satisfaction (OR = 1.426, 95% CI: 1.004-2.027, p = 0.047), as shown in Table 10. However, employment status did not independently predict satisfaction in the regression models after controlling for health and physical activity (p > 0.05).

**Table 10:** Logistic Regression Parameter Estimates Predicting Life Satisfaction from General Health Status, Physical Activity, and Employment Status Among U.S. Adults The table presents the parameter estimates from a multinomial logistic regression analysis examining life satisfaction. The reference category for life satisfaction is "Very Dissatisfied." Each intercept represents the log-odds of being in a respective category relative to the reference category when all predictors are set to zero. Significant predictors (p < .05) are indicated in the "Sig." column, with odds ratios (Exp(B)) and associated 95% confidence intervals provided for each parameter. a. The reference category is: Very Dissatisfied. b. This parameter is set to zero because it is redundant. **denotes statistical significance at the p-value of 0.05

Life Satisfaction^a^ (Dependent Variable)	Independent Variable	Regression Coefficient (B)	Std. Error	Wald	Degree of Freedom (df)	Sig. (p)	Odds Ratio (OR) (Exp(B))	95% Confidence Interval for Exp(B)	
								Lower Bound	Upper Bound
Dissatisfied	Intercept	1.909	.364	27.531	1	.000			
	Age of Sample Adult	-.004	.004	.880	1	.348	.996	.987	1.005
	General Health Status	-.004	.075	.003	1	.959	.996	.861	1.153
	Sex of Sample Adult _Female	-.381	.145	6.941	1	.008**	.683	.514	.907
	Race _A_Asian_only	-.199	.394	.254	1	.614	.820	.379	1.775
	Race _Black_African_American_only	.116	.220	.278	1	.598	1.123	.730	1.729
	Race _A_Other	.041	.261	.025	1	.874	1.042	.626	1.737
	Physical Activity_Meets_Aerobic_only	-.088	.200	.196	1	.658	.915	.619	1.354
	Physical Activity_Meets_Both_Criteria	.149	.253	.348	1	.555	1.161	.707	1.904
	Physical Activity_Meets_Strength_only	.722	.387	3.476	1	.062	2.058	.964	4.394
	Physical Activity_A_Not_Ascertained	-.105	.313	.112	1	.737	.900	.487	1.664
	[Usually Work 35+ Hours Per Week = Missing Response]	-.262	.200	1.724	1	.189	.769	.520	1.138
	[Usually Work 35+ Hours Per Week=No]	-.051	.300	.029	1	.864	.950	.528	1.709
	[Usually Work 35+ Hours Per Week=Yes]	0^b^	.	.	0	.	.	.	.
Satisfied	Intercept	1.897	.330	33.046	1	.000			
	Age of Sample Adult	.008	.004	4.059	1	.044	1.008	1.000	1.016
	General Health Status	.758	.067	127.200	1	.000**	2.134	1.871	2.435
	Sex of Sample Adult _Female	-.314	.131	5.741	1	.017**	.730	.565	.944
	Race _A_Asian_only	.617	.345	3.193	1	.074	1.853	.942	3.645
	Race _Black_African_American_only	.371	.200	3.449	1	.063	1.449	.980	2.142
	Race _A_Other	.281	.236	1.414	1	.234	1.324	.834	2.103
	Physical Activity_Meets_Aerobic_only	.154	.178	.752	1	.386	1.167	.823	1.654
	Physical Activity_Meets_Both_Criteria	.226	.230	.963	1	.326	1.253	.798	1.968
	Physical Activity_Meets_Strength_only	.807	.365	4.881	1	.027**	2.241	1.095	4.584
	Physical Activity_A_Not_Ascertained	.054	.279	.037	1	.847	1.055	.611	1.822
	[Usually Work 35+ Hours Per Week = Missing Response]	-.858	.181	22.402	1	.000**	.424	.297	.605
	[Usually Work 35+ Hours Per Week=No]	-.297	.273	1.185	1	.276	.743	.435	1.268
	[Usually Work 35+ Hours Per Week=Yes]	0^b^	.	.	0	.	.	.	.
Very Satisfied	Intercept	-1.708	.335	25.934	1	.000			
	Age of Sample Adult	.021	.004	27.880	1	.000**	1.022	1.014	1.030
	General Health Status	1.487	.068	475.311	1	.000**	4.425	3.871	5.058
	Sex of Sample Adult _Female	-.195	.132	2.176	1	.140	.823	.635	1.066
	Race _A_Asian_only	.211	.347	.371	1	.543	1.236	.625	2.441
	Race _Black_African_American_only	.238	.202	1.387	1	.239	1.268	.854	1.883
	Race _A_Other	.130	.239	.296	1	.586	1.139	.713	1.818
	Physical Activity_Meets_Aerobic_only	.355	.179	3.931	1	.047**	1.426	1.004	2.027
	Physical Activity_Meets_Both_Criteria	.492	.231	4.542	1	.033**	1.636	1.040	2.572
	Physical Activity_Meets_Strength_only	.885	.367	5.817	1	.016**	2.423	1.180	4.974
	Physical Activity_A_Not_Ascertained	.038	.283	.018	1	.892	1.039	.597	1.808
	[Usually Work 35+ Hours Per Week = Missing Response]	-.878	.182	23.143	1	.000**	.416	.291	.594
	[Usually Work 35+ Hours Per Week=No]	-.368	.274	1.799	1	.180	.692	.405	1.185
	[Usually Work 35+ Hours Per Week=Yes]	0^b^	.	.	0	.	.	.	.

## Discussion

The findings of this study underscore the critical roles of health status and physical activity in shaping life satisfaction among U.S. adults, while employment status exhibited a comparatively weaker influence. As shown in Table 2, self-reported health status demonstrated a robust positive correlation with life satisfaction (γ = 0.514, p < 0.001), aligning with prior research emphasizing the centrality of perceived health in subjective well-being [6,7,25]. The strong association between health and life satisfaction aligns with an earlier study [26], which found that both positive and negative affect mediate the relationship between self-rated health and well-being. This underscores the bidirectional nature of health and satisfaction, where mental states amplify perceived health outcomes. Barger et al. [6] identified self-rated health as the strongest predictor of life satisfaction, even after adjusting for socioeconomic factors. Physical activity’s role in enhancing satisfaction further supports An et al. [27], who linked exercise to improved autonomy and social engagement among older adults.

Individuals reporting excellent health were significantly more likely to express high life satisfaction (68.3% very satisfied), whereas those in poor health predominantly reported dissatisfaction (32.1%), as in Table 3. This trend mirrors global patterns observed in populations with chronic health conditions and limited healthcare access [1,25,26]. The strong association between health and life satisfaction highlights the need for preventive care and accessible health services to mitigate disparities, particularly among marginalized groups facing systemic barriers [3,4,28]. Intersectionality frameworks [3,4,16] further elucidate how overlapping identities - such as race, gender, and socioeconomic status - compound barriers to healthcare and recreational resources, exacerbating inequities in life satisfaction. For instance, racial minorities often face higher rates of chronic conditions and reduced access to safe exercise environments, which disproportionately diminish their well-being [4,14].

Physical activity emerged as another pivotal determinant of life satisfaction, with adherence to both aerobic and strength-training guidelines correlating with higher satisfaction levels (Table 6). Participants meeting both criteria reported 54.5% were very satisfied, compared to 37.9% among those meeting neither. These results resonate with studies linking physical activity to improved mental health and resilience [14,27,29]. The graded relationship between activity intensity and satisfaction suggests that public health initiatives promoting comprehensive exercise regimens could yield significant well-being benefits [30]. However, structural barriers - such as unsafe neighbourhoods, financial constraints, and lack of culturally tailored programs - often limit physical activity participation in low-income and minority communities. Addressing these disparities requires policies that prioritize equitable access to recreational infrastructure, such as parks and community centres, particularly in underserved areas [14,27].

Contrastingly, employment status exhibited a modest association with life satisfaction (χ² = 9.988, p = 0.019) as shown in Table 8, with only a 1.2% difference in very satisfied responses between those working ≥35 hours/week and others (Table 9). This weak linkage challenges traditional assumptions about employment as a primary driver of well-being, suggesting that job quality and workplace environments may outweigh mere hours worked [5,10,11]. Notably, individuals with missing employment data reported markedly lower satisfaction, potentially reflecting underlying stressors such as job insecurity or informal work conditions. These findings align with studies where unstable employment eroded life satisfaction [10,11], emphasizing the need to address broader socioeconomic determinants beyond employment metrics. For example, Chen and Hou [11] highlighted that unemployment’s negative impact on life satisfaction persists across nations, underscoring the role of financial strain and social exclusion. Workplace interventions focusing on mental health support, flexible schedules, and equitable wages may thus be more impactful than focusing solely on employment rates [5,28].

The regression analysis (Table 10) further clarified these relationships, revealing that health status and physical activity accounted for significant variance in well-being [7,14]. Age and gender also played significant roles, with older adults and females reporting higher satisfaction - a finding consistent with literature on aging and resilience [21,28]. However, the intersectional lens revealed nuanced disparities: while race/ethnicity did not independently predict satisfaction, systemic inequities in healthcare access and physical activity opportunities persisted among minority groups [3,4,16]. For instance, women may face gendered expectations that limit their leisure time for exercise, while older adults from marginalized backgrounds often encounter ageism compounded by racial discrimination. These results underscore the importance of tailored interventions addressing overlapping social identities to reduce well-being gaps [3,4].

Demographic factors further modulated these outcomes. Younger participants reported higher physical health scores (β = -0.516, p < 0.001), likely due to greater adaptability and fewer chronic conditions, whereas older adults’ life satisfaction may reflect accumulated coping strategies [27]. Gender disparities in mental health scores, with females outperforming males (Mental Component Score (MCS): p = 0.029), contrast with refugee studies where social support programs improved women’s outcomes [21]. This divergence suggests that cultural and contextual factors, including access to gender-specific health resources, critically shape well-being trajectories [3,21]. For example, Cherepanov et al. [21] noted that women’s higher health-related quality of life in the U.S. may stem from greater healthcare utilization, whereas systemic barriers in other contexts reverse this trend.

Policy implications are profound. Prioritizing healthcare access, particularly preventive services, could alleviate the burden of chronic conditions exacerbating dissatisfaction [26]. Expanding community recreational infrastructure, especially in underserved areas, may enhance physical activity participation [14,27]. Workplace reforms fostering job security, flexibility, and mental health support are equally vital [5,11,28]. For marginalized populations, intersectionality-driven policies, such as linguistically accessible health education and culturally competent care, are essential to address compounded barriers [3,4]. For instance, the National Health Interview Survey’s findings [17] on demographic trends call for targeted outreach to aging populations, ensuring that health interventions accommodate mobility limitations and social isolation common in later life. Further studies should also investigate the effectiveness of tailored interventions - such as personalized fitness regimens and mental health support - for diverse demographic groups to optimize well-being outcomes. By integrating these findings into public health and organizational policies, stakeholders can better address the multifaceted drivers of life satisfaction and improve quality of life across communities.

This study has several limitations that should be acknowledged. First, the cross-sectional design precludes causal inferences regarding the relationships between health status, physical activity, employment status, and life satisfaction. Temporal dynamics remain unresolved, as the data capture associations at a single time point. Longitudinal investigations are necessary to establish whether improved health and physical activity precede higher life satisfaction or vice versa [7]. Second, self-reported measures of health and physical activity may introduce biases, such as social desirability or recall inaccuracies. While prior research supports the validity of self-reported health metrics through their alignment with objective measures (e.g., clinical assessments, accelerometer data) [24], subjective reporting risks overestimating adherence to activity guidelines or underreporting health conditions due to stigma. Third, the use of broad demographic categories (e.g., aggregated racial/ethnic groups, age dichotomized as 18-84 vs. 85+) may mask heterogeneity within subgroups [3]. For instance, classifications such as “Asian” or “Black/African American” fail to account for cultural, socioeconomic, or immigration-related variations that could differentially influence health behaviours and well-being. Similarly, collapsing age into binary ranges overlooks nuanced life satisfaction trajectories across adulthood. Complementary qualitative methodologies, such as in-depth interviews, could elucidate how intersecting identities and lived experiences shape these relationships [4,25]. Finally, while the analysis adjusted for key demographic variables, unmeasured confounders, including socioeconomic status, mental health history, social support networks, and healthcare access, may influence both predictors (e.g., health status) and outcomes (life satisfaction). Future studies should incorporate these variables to refine predictive models and enhance explanatory power.

Despite these limitations, this study has several notable strengths, including its large, nationally representative sample and comprehensive analysis of multiple determinants of life satisfaction. The use of robust statistical methods and validated measures ensures the reliability of our findings, while the intersectional lens provides valuable insights into disparities across demographic groups. These strengths underscore the study’s contribution to public health research and its potential to inform policies aimed at enhancing well-being.

## Conclusions

This study highlights significant associations between self-reported health, physical activity, employment status, and life satisfaction among U.S. adults. The results indicate that individuals reporting better health and those who meet both aerobic and strength-training activity guidelines tend to experience higher life satisfaction, underscoring the value of comprehensive health promotion initiatives. Conversely, the relatively weaker association observed with employment status suggests that workplace interventions should extend beyond adjustments to work hours and instead emphasize broader well-being strategies.

Given these results, public health interventions should emphasize accessible healthcare, community-based physical activity programs, and workplace wellness policies to enhance overall life satisfaction. Future research should employ longitudinal designs to establish causality and explore additional moderators, such as socioeconomic status and mental health, while qualitative methodologies could provide deeper insights into subgroup variations. Targeted interventions addressing health behaviors and social determinants of well-being are essential for fostering healthier, more satisfied populations. Policymakers, healthcare providers, and employers must collaborate to implement evidence-based strategies that bridge gaps in health equity and promote sustainable lifestyle improvements.
